# Chicken IgY reduces the risk of *Pseudomonas aeruginosa* urinary tract infections in a murine model

**DOI:** 10.3389/fmicb.2022.988386

**Published:** 2022-09-09

**Authors:** Franziska A. Schwartz, Lars Christophersen, Kim Thomsen, Sarah Baekdal, Maria Pals Bendixen, Mette Jørgensen, Ida Kirstine Bull Rasmussen, Anne Sofie Laulund, Niels Høiby, Claus Moser

**Affiliations:** ^1^Department of Clinical Microbiology, Rigshospitalet, Copenhagen University Hospital, København, Denmark; ^2^Department of Immunology and Microbiology, Costerton Biofilm Center, University of Copenhagen, København, Denmark

**Keywords:** prophylaxis, IgY, urinary tract infection, biofilm, antibiotic resistance, *Pseudomonas aeruginosa*, spinal cord injury, inflammation

## Abstract

**Introduction:**

Urinary tract infections (UTIs) with *Pseudomonas aeruginosa* are a severe problem in disposed patients in modern healthcare. *Pseudomonas aeruginosa* establishes recalcitrant biofilm infections and can develop antibiotic resistance. Gargling with avian egg yolk anti-*Pseudomonas* antibodies (IgY) has shown clinical effect in preventing onset of chronic *P. aeruginosa* lung infections in patients with cystic fibrosis (CF). Therefore, we speculated whether passive intravesically administered IgY immunotherapy could be a novel strategy against *P. aeruginosa* UTIs.

**Aim:**

To evaluate if prophylactic repurposing of anti-*Pseudomonas* IgY can prevent UTIs with *P. aeruginosa* in a UTI mouse model.

**Materials and methods:**

*In vitro*, *P. aeruginosa* (PAO1 and PAO3) was mixed with increasing concentrations of specific anti-*Pseudomonas* IgY (sIgY) or non-specific control IgY (cIgY) and/or freshly isolated human neutrophils. Bacterial growth was evaluated by the optical density at 600 nm. *In vivo*, *via* a temporary transurethral catheter, 10-week-old female Balb/c mice were intravesically infected with 50 ml of a bacterial suspension and sIgY, cIgY, or isotonic NaCl. IgY and NaCl were either co-instilled with the bacteria, or instilled prophylactically, 30 min prior to infection. The animals were euthanized 20 h after infection. Vesical bacteriology was quantified, and cytokine expression in the bladder homogenate was measured by multiplex cytokine assay.

**Results:**

*In vitro*, sIgY concentrations above 2.5% reduced bacterial growth in a dose-dependent manner. *In vivo*, a UTI lasting for minimum 7 days was established by installing 5 × 10^6^ colony-forming units (CFU) of *P. aeruginosa* PAO1. sIgY reduced vesical bacterial load if co-installed with *P. aeruginosa* PAO1. Prophylactic sIgY and cIgY reduced bacterial load when compared to isotonic NaCl. CXCL2 and G-CSF were both increased in infected bladders compared to non-infected controls which had non-detectable levels. Co-installation of sIgY and bacteria nearly completely inhibited the inflammatory response. However, the cytokine levels in the bladder did not change after prophylactic administration of sIgY or cIgY.

**Conclusion:**

Prophylactic sIgY significantly reduces the amount of bacteria in the bladder in a mouse model of *P. aeruginosa* cystitis and may serve as a novel non-antibiotic strategy in preventing *P. aeruginosa* UTIs.

## Introduction

Urinary tract infections (UTIs) are responsible for numerous contacts to the health care system and a major cause of antibiotic consumption in adult patients (Stamm and Norrby, 2001; Litwin et al., 2005). Urinary tract infections can affect the bladder (cystitis), kidneys (pyelonephritis), and may progress to the bloodstream resulting in sepsis (Foxman, 2014). Uncomplicated UTI occur in non-pregnant, healthy, adult women, and have a low risk of complications. Complicated UTI occur in men, children and pregnant women, and are frequent amongst hospitalized, critically ill and immunocompromised patients, and in patients with compromised bladder emptying, e.g., spinal cord injured (SCI) patients (Foxman, 2003; Tofte et al., 2017). Additional factors increasing the risk of developing UTIs are frequent and include diabetes mellitus, age, anatomical/physiological abnormalities of the urinary tract, and urolithiasis or urinary catheter use (Boyko et al., 2002; Foxman, 2014; Nitzan et al., 2015).

While uropathogenic *Escherichia coli* is reported as the microbial etiology causing 70–80% of uncomplicated UTIs (Foxman, 2014), *Pseudomonas aeruginosa* causes around 10% of nosocomial UTIs which are often difficult to treat due to the bacteria’s intrinsic antibiotic resistance mechanisms, and the inherent ability to acquire further antibiotic resistance (Cek et al., 2014; Lamas Ferreiro et al., 2017; Ciofu and Tolker-Nielsen, 2019). The opportunistic pathogen is known for its ability to form biofilms which are bacterial aggregates surrounded by a self-produced exopolysaccharide matrix (Sharma et al., 2014). Nosocomial UTI with *P. aeruginosa* are especially common in male patients and in patients previously receiving antibiotic treatment (Djordjevic et al., 2013). *Pseudomonas aeruginosa* can form UTI in relation to the presence of external and internal urinary tract catheters or nephrostomy catheters (Tofte et al., 2017). However, the bacteria can also establish recurrent or persistent infections without a permanent catheter as in the case of intermittent catheterization (Esclarín De Ruz et al., 2000). Considering the ability of *P. aeruginosa* to establish chronic recalcitrant biofilms and subsequent antibiotic resistance, *P. aeruginosa* UTI constitute a substantially larger healthcare problem than the prevalence of 10% indicates. High 30 and 90 days mortalities of 17.7 and 33.9% have been reported in hospitalized patients with *P. aeruginosa* UTI (Lamas Ferreiro et al., 2017). In addition, patients with complicated UTI are at risk of developing recurring infections, which have a strong adverse effect on the patient’s quality of life. Urinary tract infection in general, and *P. aeruginosa* UTI in particular, are a considerable economic burden to the healthcare system (Foxman, 2003; Simmering et al., 2017). Therefore, it is important to identify approaches to prevent UTI, especially in high-risk patients.

Passive immunotherapy with anti-*Pseudomonas* egg yolk antibodies is reported to be a promising prophylactic treatment strategy against lung colonization with *P. aeruginosa* in cystic fibrosis (CF) airways (Nilsson et al., 2008; Thomsen et al., 2016b). IgY is a serum immunoglobulin in chicken that is synthesized by the hen and accumulates in the egg yolk (Patterson et al., 1962; Rose et al., 1974; Warr et al., 1995). It is the avian homolog of mammalian IgG and provides the offspring with a humoral immune response after hatching. Similar to mammalian IgG, IgY is composed of two heavy chains and two light chains containing the variable region. IgY is less flexible than IgG due to the missing hinge region, which in turn protects the antibody from degradation and fragmentation to a higher degree and provides a subsequent long half-life of months at room temperature and of years at 4°C (Pereira et al., 2020).

Anti-*Pseudomonas* IgY can rapidly and effortlessly be produced by immunizing hens with specific antigens and then isolating the antibodies from the egg yolk (Bhanushali et al., 1994).

The antibacterial effect of IgY is known to be multifactorial and has been studied extensively in different *in vitro* and *in vivo* infection models using different bacterial species. The beneficial mechanism is believed to be a combination of host-immune modulation (Li et al., 2016), bacterial growth inhibition (Lee et al., 2002; Zhen et al., 2008; Wang et al., 2019), blocking bacterial adherence to host tissues (Jin et al., 1998; Wang et al., 2011; Pizarro-Guajardo et al., 2017), pathogen agglutination (Lee et al., 2002; Thomsen et al., 2015), bacterial opsonization, thereby facilitating phagocytosis (Zhen et al., 2008; Thomsen et al., 2015), and neutralizing bacterial toxins (Wang et al., 2011). Recently, we revealed an additive antibiotic-enhancing effect of sIgY in combination with azithromycin in an experimental mouse model of chronic *P. aeruginosa* lung infection (Thomsen et al., 2021).

In this work, we aimed at evaluating the potential of prophylactic IgY as a non-antibiotic strategy to prevent *P. aeruginosa* colonization and subsequent infection of the urinary tract. Such a strategy can potentially decrease antibiotic use and the risk of antibiotic resistance development. This approach could be particularly valuable for intensive care unit (ICU) or renal-transplanted patients at high risk of developing UTI with *P. aeruginosa*, or for SCI patients with impaired micturition using intermittent catheterization.

## Materials and methods

### IgY

The IgY antibodies were purchased from IgY Lab Systems AB, Vittinge, Sweden. They stem from White leghorn chickens that were either naïve (non-specific control IgY, cIgY) or immunized with six different *P. aeruginosa* strains (O:1, O:3, O:5, O:6, O:9, and O:11) (Thomsen et al., 2015) (specific anti-*Pseudomonas* IgY, sIgY). The cIgY served as an isotype control. The IgY undiluted stock concentration for both batches was 20 mg/mL.

### *In vitro* growth retardation assay

Bacterial overnight cultures of *P. aeruginosa* PAO1 (serotype O:5) and a *P. aeruginosa* strain of serotype O:3 were incubated in lysogeny broth (LB [SSC Panum, Copenhagen, Denmark]). The two strains were also included in our previous *in vitro* IgY studies (Jin et al., 1998; Thomsen et al., 2016b). Thirty minutes prior to the assay start, the culture was centrifuged [10 min, 5,000 RPM (4K15, rotor 11,140, Merck)], resuspended to the overnight culture concentration in Krebs-Ringer Buffer (KRb) (containing 0.2% glucose [SSC Panum, Copenhagen, Denmark]), mixed with 500 μL human AB + serum and opsonized for 15 min at 37°C. The solution was then diluted 60-fold in LB.

sIgY and cIgY were used undiluted (100%, 20 mg/mL) or diluted in LB to reach 50% (10 mg/mL), 25% (5 mg/mL), 12.5% (2.5 mg/mL), 6% (1.2 mg/mL), 3% (0.6 mg/mL), and 1.5% (0.3 mg/mL).

Human polymorphonuclear neutrophils (PMN) were isolated from healthy human volunteers by venous puncture. The blood was collected in Vacutainers containing 3.2% buffered citrate (BD, New Jersey, NJ, United States). Per 4 mL blood, 1 mL 5% dextran (SSC Panum, Copenhagen, Denmark) was added and the fractions were allowed to separate for 30–60 min at room temperature. The upper fraction was overlaid over 4 mL LymphoprepTM (Stemcell Technologies, Cambridge, United Kingdom). The samples were centrifuged (2,200 RPM, room temperature, 15 min), erythrocytes were lysed for 30–40 s with 0.2% saline, quenched with 1.6% saline, followed by centrifugation (1,800 RPM, room temperature, 10 min). The erythrocyte lysis was repeated once. The cell pellet was resolved in KRb with 0.2% glucose. The neutrophils were adjusted to 3.5 × 10^6^ cells/mL by measuring in a NucleoCounter (Chemometec, Allerød, Denmark).

The assay was carried out in a 96-well optical plate (Nunc, Thermo Scientific, Waltham, MA, United States). Sixty μL [1 × 10^6^ colony-forming units (CFU)] of the bacteria, 40 μL of the diluted sIgY or cIgY and 100 μL (3.5 × 10^5^ cells) of the neutrophil suspension were mixed. Wells without IgY and/or neutrophils served as growth controls.

The plate was read in a multiplate reader at 600 nm (Multiscan FC Microplate Reader, Thermo Scientific, Waltham, MA, United States) at the start of the experiment and hourly up to 6 h. The start reading was subtracted from the optical densities (OD) at the different timepoints. The obtained change in OD (dOD) served as an estimate for differences in the bacterial growth.

### Murine urinary tract infection

Over the course of the three experiments, a total of 95 10-week-old, female BALB/c mice were included (Janvier Labs, Le Genest-Saint-Isle, France) and acclimatized for 1 week prior to experiments. BALB/c mice were chosen, as they previously proved most susceptible in our airway infection model (Moser et al., 1997). Female mice were used exclusively, as they can more easily be housed in bigger groups and are furthermore easier to infect intraurethrally. The anesthesia protocol was adjusted to 12-week-old mice. All mouse experiments were conducted in accordance with procedures approved by the Danish Animal welfare legislation (Permission nr. 2018-15-0201-01516). All animals had free access to water and chow. Prior to all procedures, mice were anaesthetized with 250 μL of a mixture of one part Hypnorm [final concentrations per mouse: 19.7 μg Fentanylcitrate, 625 μg Fluanisone, and 31.25 μg Methylparahydroxybenzoate (Panum, Department for Experimental Medicine, Copenhagen, Denmark)], one part Midazolam (312.5 μg/mouse [5 mg/mL stock solution, Hameln pharma, Hameln, Germany]) and two parts sterile water for injection. The anesthesia dose was chosen higher than the minimal necessary dose, as that was found to reduce voiding during the anesthesia period. The mice were randomized into the treatment groups in experiments 2 and 3. The infection with bacteria and the treatment with IgY were installed intravesically through a temporary transurethral catheter (BD Neoflon Pro, 26 GA, 0.6 × 19 mm; BD Biosciences, Plymouth, United Kingdom). The overall volume injected per day was 50 μL. Mice that visibly urinated in the 30 min observation period after the procedure were registered but underwent the same evaluation as non-voiding mice. However, the results from voiding mice were excluded from the final analysis.

#### Experiment 1

Objective: To establish a prolonged *P. aeruginosa* UTI without a permanent transurethral catheter.

Alginate embedded bacteria were prepared as described previously (Christophersen et al., 2012). In short, alginate (Protanal LF 10/60, IMCD, Helsingør, Denmark) was adjusted to a concentration of 1% in 0.9% saline and sterilized by heating (95°C, 15 min). A bacterial overnight culture of *P. aeruginosa* in LB medium was diluted 1:20 in the alginate solution, transferred into a 20 mL syringe and placed in a syringe pump (Graseby 3,100, Ardus Medical Inc., Watford, United Kingdom). A gelling bath containing 0.1 M Tris-HCL and 12.5 mM CaCl_2_ was prepared on top of a magnetic stirrer (lab disc, VWR, Rødovre, Denmark). A plastic tube with a nozzle (0.25 mm, Nisco Engineering AG, Zurich, Switzerland) was installed 8 cm above the surface of the gelling solution. The bacteria-alginate solution was extruded through a bead generator (Encapsulation Unit Var J30, Nisco Engineering AG, Zurich, Switzerland) connected to the tube at a flow rate of 40 mL/h. The gelled beads remained in the gelling bath for 1 h, followed by two washings in 0.9% saline containing CaCl_2_.

Alginate-embedded bacteria and planktonic bacteria were both adjusted to 1 × 10^8^ CFU/mL in saline prior to infection.

Thirty-six mice were anaesthetized and randomized to receive 50 μL of either alginate-embedded bacteria (18 mice) or planktonic bacteria (18 mice). Six mice of both groups were then euthanized on day one, three, or seven after the established infection. Blood (BD Microtainer, SST tubes, BD, Plymouth, United Kingdom), kidneys and bladder were collected. The organs were kept in 1 mL 0.9% saline on ice until all mice were euthanized. The tissue samples were then homogenized (SilentCrusher M, Heidolph Instruments, Schwabach, Germany) for 30 s. Afterward, the samples were serially diluted in 0.9% saline and plated on Lactose agar plates (SSI diagnostics, Hillerød, Denmark).

#### Experiment 2

Objective: To test the immediate IgY effect on bacterial viability in two bacterial infectious doses.

Two days prior to the experiment, a PAO1 overnight culture was prepared and incubated overnight. On the day before the experiment, the culture was washed in PBS, diluted and plated on lactose agar plates. The culture was then stored at 5°C until use. On the day of the experiment, the CFU were counted and the bacterial culture was diluted in saline to reach concentrations of 2 × 10^9^ CFU/mL and 2 × 10^7^ CFU/mL. The final inoculum concentration was confirmed by plating.

The IgY solution was diluted to reach 40% (8 mg/mL) in saline, so the final injected concentration would be 20% (4 mg/mL). This concentration was found to be promising according to the *in vitro* experiments.

The bacteria and the sIgY solutions (or saline for the untreated controls) were then mixed to equal parts to achieve the following mixtures:

(i) 1 × 10^9^ CFU/mL + 20% sIgY
(ii) 1 × 10^9^ CFU/mL − sIgY
(iii) 1 × 10^7^ CFU/mL + 20% sIgY
(iv) 1 × 10^7^ CFU/mL − sIgY

Thirty-two mice were anaesthetized, randomized into the four treatment groups, and infected intravesically through a temporary transurethral catheter with 50 μL of the different solution mixtures reaching: (i) 5 × 10^7^ CFU/mouse, treated with 20% (0.2 mg/mouse) IgY; (ii) 5 × 10^7^ CFU/mouse, placebo treated with saline; (iii) 5 × 10^5^ CFU/mouse, treated with 20% (0.2 mg/mouse) IgY; (iv) 5 × 10^5^ CFU/mouse, placebo treated with saline. After the intravesical bacterial installation, all mice were placed on their back on a heated Styrofoam plate for 30 min for observation for bladder voiding.

Twenty hours after the infection, all mice were euthanized and blood was obtained as described previously. The bladders were removed into 2 mL microcentrifuge tubes (Nerbe plus, Winsen/Luhe, Germany) containing five glass beads and 500 μL saline. The bladders were then homogenized (TissueLyser II; Qiagen, Copenhagen, Denmark) for 30 min at a frequency of 30/s.

The bladder homogenates were then diluted in saline, plated on lactose agar plates (SSI Diagnostics, Hillerød, Denmark), incubated overnight and the CFU were counted the next day.

#### Experiment 3

Objective: to investigate the prophylactic potential of IgY.

The bacterial preparation was performed similarly to experiment 2. The concentration was adjusted to 2 × 10^7^ CFU/mL. The sIgY and cIgY solutions were diluted to reach 40% (8 mg/mL) in saline.

Twenty-seven mice were anaesthetized and randomized into groups of nine mice each, and infected intravesically though a temporary transurethral catheter with 25 μL of either sIgY (0.2 mg/mouse) (i), cIgY (0.2 mg/mouse) (ii) or saline (iii). The temporary catheters were removed, the mice were placed on their backs and the treatment was left in place for 30 min under constant observation. Afterward, all mice were catheterized again and infected with 25 μL of the bacterial solution, achieving an infectious load of 5 × 10^5^ CFU/mouse. Again, all mice were observed for 30 min for bladder voiding following the procedure.

After 20 h, the mice were euthanized, and blood and organs were collected as described in experiment 2. The supernatant of the bladder homogenate was collected, sterile filtered (0.22 μm; FilterBio, Nantong City, China) and stored at −80°C until use.

### Luminex

Luminex™ multiplex analysis (Mouse Magnetic Luminex Assays, R&D systems, Abingdon, United Kingdom) was performed in duplicates with the sterile filtered bladder homogenates of experiment 2 (infection groups III and IV) and experiment 3 according to the manufacturer’s instructions. The samples were diluted two times. Plates were read in a Luminex 200™ Platform (Luminex Corp., Austin, TX, United States). The following analytes were measured: CXCL1, G-CSF, IL-6, CXCL2, and IL-1b.

## Results

### Specific IgY can retard PAO1 growth

This assay was performed to determine the IgY concentrations suitable for the *in vivo* use.

**FIGURE 1:**
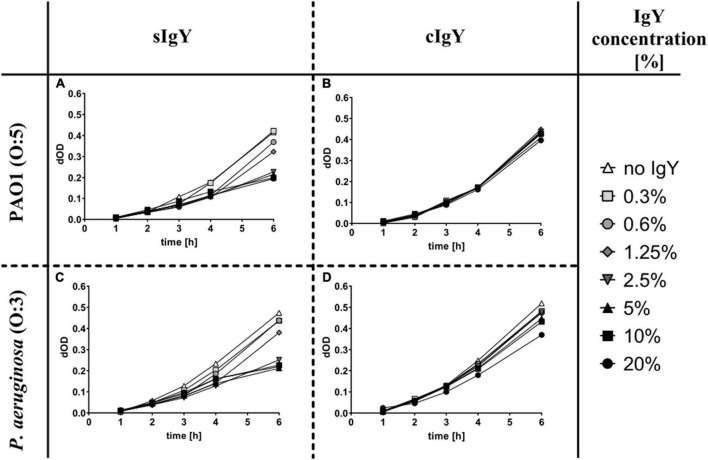
Growth retarding effect of specific anti-*Pseudomonas* IgY *in vitro*. *Pseudomonas aeruginosa* PAO1 **(A,B)** and PAO3 **(C,D)** were grown in lysogeny broth (LB) overnight, centrifuged, resuspended in Krebs-Ringer buffer (KRb), opsonized with human AB^+^ serum and then diluted 60 times in LB to reach a final well concentration of approx. 5 × 10^6^ CFU/mL. Specific **(A,C)** and non-specific **(B,D)** IgY were diluted to the desired concentrations (final well concentrations between 20 and 0.3%). Human PMNs were isolated from healthy volunteers and cell counts were adjusted to reach final well concentrations of 1.75 × 10^6^ cells/mL. The 96-well plates were read hourly in a multiplate reader at 600 nm for up to 6 h. The start reading was subtracted from the following readings as the background. Displayed are the changes in OD (dOD), corresponding to the bacterial growth over time.

Observing the growth of *P. aeruginosa via* the optical density (600 nm) can give an idea of growth retarding effects of the treatments. For PAO1, the cIgY did not alter the growth significantly, while the sIgY in concentrations of 2.5% (0.5 mg/mL) and above had a dose-dependent growth retarding effect. A similar effect of sIgY was observed for serotype O:3 strain, although, for the cIgY in a concentration of 20% (4 mg/mL), a marginal growth retarding effect was observed (Figure 1).

### Planktonic bacteria establish a persistent urinary tract infection independent of alginate embedment and permanent catheter

From our experience in a chronic murine lung and wound infection models, we hypothesized that alginate embedded bacteria might establish a more stable UTI than planktonic bacteria, as they better resemble biofilm-characteristics. To compare the potential of planktonic and alginate-embedded bacteria to establish a stable, long-term cystitis, 5 × 10^6^ CFU P. aeruginosa were installed through a temporary transurethral catheter into the bladder of each mouse.

**FIGURE 2:**
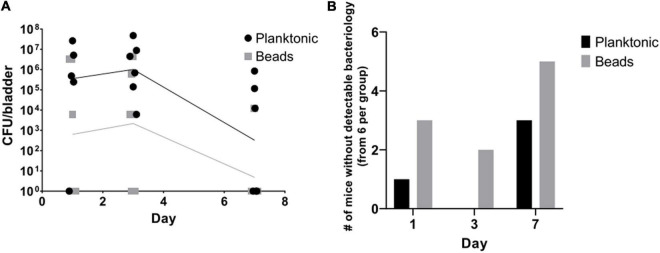
Planktonic bacteria achieve more stable infections than alginate-embedded (beads) bacteria *in vivo*. Alginate embedded bacteria were produced prior to the procedure as described in the methods. Planktonic and embedded bacteria were adjusted to 1 × 10^8^ CFU/mL on the day of experiment start. Thirty-six 10-week old female Balb/c mice were anesthetized and randomized in equal groups receiving 50 μL of either planktonic or alginate embedded bacteria. On days one, three and seven, six mice from each group were euthanized. Blood, kidneys, and bladders were harvested. The organs were homogenized and quantitative bacteriology was evaluated the next day. Displayed are the CFU (lines connect geometric means) detected in the bladders of the mice **(A)** and the amount of mice without any detectable bacteria in the bladder **(B)**.

We observed that planktonic bacteria established UTI in the majority of the mice (5 of 6 mice on day 1; 6 of 6 mice on day 3, and 3 of 6 mice on day 7). Mice infected with alginate-embedded bacteria had a lower success rate (3 of 6 mice on day 1, 4 of 6 on day 3, and 1 of 6 mice on day 7) (Figures 2A,B). In addition, the mice injected with planktonic bacteria generally had a non-significantly higher bacterial load in the bladder than the mice injected with the alginate beads (Figure 2). In a series of follow-up experiments (data not shown), the infection dose was increased to 5 × 10^7^ CFU/mouse, resulting in culturable *P. aeruginosa* in the urinary bladder up to day 13 in 7 out of 8 mice, confirming the model as a persistent infection. Subsequently, the intravesical infection was decreased to 5 × 10^5^ CFU/mouse achieving a 100% success rate for the instillation of a stable UTI after 20 h (data not shown).

### Co-administered specific anti-*Pseudomonas* IgY reduces PAO1 bacteriuria *in vivo*

**FIGURE 3:**
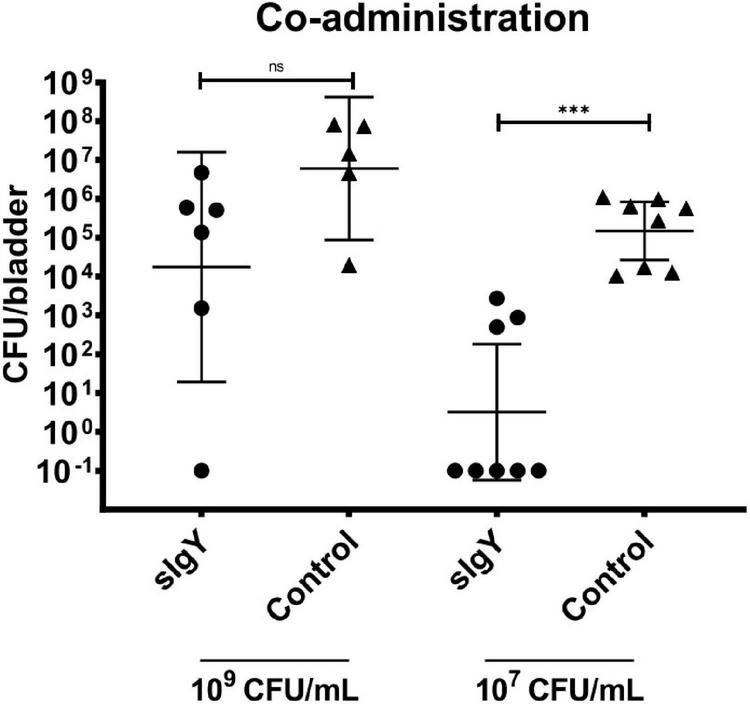
Co-administration of IgY during infection lowers bladder bacteriology *in vivo*. A bacterial overnight culture of PAO1 was adjusted to either 2 × 10^9^ or 2 × 10^7^ CFU/mL in saline. IgY was diluted to reach 40% in saline. Bacteria and IgY or saline (control group) were then mixed to equal parts immediately prior to experiment start. Thirty-two 10-week-old female Balb/c mice were randomized into the four groups, anesthetized, intravesically infected with 50 μL of the bacteria mixed with IgY or saline and observed for 30 min after infection to exclude mice that were voiding during this period. Twenty hours after infection, all mice were euthanized. Blood, kidneys, and bladders were harvested. The organs were homogenized and quantitative bacteriology was evaluated the next day. Displayed are the CFU (geometric mean with 95% confidence interval) detected in the bladders of all not excluded mice. Statistically significant differences were evaluated by Mann–Whitney tests (^***^p ≤ 0.001). ns, non-significant.

One mouse in group (ii) died due to the anesthesia. In the high exposure group, in two sIgY and two non-treated mice, urine void was observed, and the mice were excluded from further analysis. When the mice were infected with a mixture of *P. aeruginosa* 5 × 10^7^ CFU or 5 × 10^5^ CFU and 20% (0.2 mg) IgY or saline as controls, we observed that most sIgY treated mice had a lower bacterial load in the bladder than the control mice [mean: 1.44 × 10^7^ vs. 3.23 × 10^5^ (high dose, *p* = 0.08), and 4.23 × 10^5^ vs. 0 (low dose, *p* = 0.0002)]. All mice in the saline treated group had bacteriuria, whereas one of the mice with higher infectious load (n.s), and five mice with the lower infectious load had no detectable bacteria in the bladder (*p* = 0.007; Figure 3).

### Specific anti-*Pseudomonas* IgY prophylaxis reduces PAO1 bacteriuria *in vivo*

**FIGURE 4:**
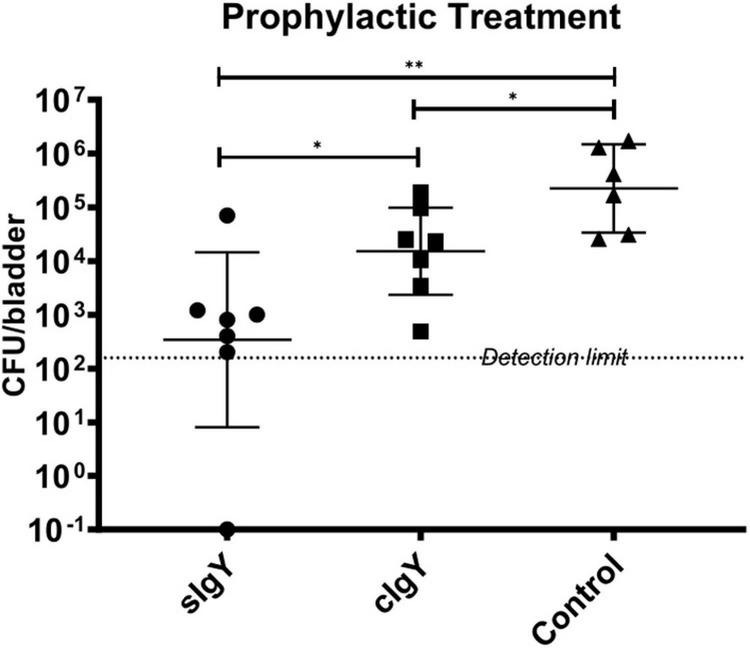
Prophylactic treatment with both specific and control IgY reduces bladder bacteriology *in vivo*. A bacterial overnight culture of PAO1 was adjusted to 2 × 10^7^ CFU/mL in saline. sIgY and cIgY were diluted to reach 40% in saline. Twenty-seven 10-week-old female Balb/c mice were randomized into the three groups, anesthetized, and 25 μL of IgY was installed intravesically. After 30 min, 25 μL of the bacterial solution was injected intravesically. The mice were observed for another 30 min after infection to exclude mice that were voiding during this period. Twenty hours after infection, all mice were euthanized. Blood, kidneys, and bladders were harvested. The organs were homogenized and quantitative bacteriology was evaluated the next day. Displayed are the CFU (geometric mean with 95% confidence interval) detected in the bladders of all not excluded mice. Statistically significant differences were evaluated by Mann–Whitney tests (*p ≤ 0.05, ^**^p ≤ 0.01).

We excluded two mice of each IgY group and three mice of the saline group as they urinated within 30 min after prophylaxis or infection. When either sIgY or cIgY were administered intravesically 30 min prior to infection with *P. aeruginosa* PAO1, the bacterial load in the bladder was significantly reduced compared to the saline control group [2.91 × 10^5^ to 8.1 × 10^2^ (sIgY; *p* = 0.005) or to 2.31 × 10^4^ (cIgY; *p* = 0.022)]. Furthermore, the mice receiving prophylaxis with sIgY had decreased bacterial vesical load as compared to the mice receiving cIgY prophylaxis (*p* = 0.038; Figure 4).

### CXCL2 and G-CSF elevated in infected bladders

Cytokine measurements revealed non-detectable levels of G-CSF, IL-6, IL-1β, and CXCL2 in all non-infected, healthy control bladders, except in one mouse, where 5 pg/ml of CXCL2 was measured. Increased cytokine levels were observed in varying levels in the bladders of the infected, but untreated mice: in one mouse for IL-1β (ns), three mice for IL-6 (*p* < 0.05), five mice for CXCL2 (*p* < 0.025) and all six mice for G-CSF (*p* = 0.0005). The levels of the chemokine CXCL2 and the neutrophil mobilizer G-CSF were increased compared to non-infected bladders (*p* = 0.05 and *p* < 0.0025; Table 1).

**TABLE 1:** *Pseudomonas aeruginosa* PAO1 infection increases inflammatory cytokines in bladder.

	Concentration (pg/mL)			
	
	IL-1β	IL-6	CXCL2	G-CSF
PAO1 (*n* = 9)	0 (0–110)	1 (0–30)	4 (0–60)	7 (4–14)
Non-infected (*n* = 6)	0 (0–0)	0 (0–0)	0 (0–5)	0 (0–0)
Mann–Whitney	*p* = 0.99	*p* = 0.18	*p* = 0.054	*p* = 0.0022

Data are presented as median (range).

Specific anti-*Pseudomonas* IgY almost completely abolished an inflammatory cytokine response when co-administered with PAO1 intravesically (Table 2). Compared to the saline control mice, co-instillation of sIgY with the lower bacterial challenge significantly reduced the detected cytokine levels. [IL-1β: 0 (0–0) vs. 66 (0–117) pg/ml, *p* = 0.0014: IL-6: 0 (0–0) vs. 11.5 (0–92) pg/ml, *p* = 0.0014; CXCL2: 0 (0–2) vs. 15.5 (10–313) pg/ml, *p* = 0.0002; G-CSF: 1.5 (1–4) vs. 22.5 (6–164) pg/ml, *p* = 0.0002].

**TABLE 2:** Co-instillation of sIgY with bacteria reduces inflammatory cytokines in the bladder.

	Concentration (pg/mL)			
	
	IL-1β	IL-6	CXCL2	G-CSF
sIgY (*n* = 8)	0 (0–0)	0 (0–0)	0 (0–2)	1.5 (1–4)
Saline (*n* = 8)	66 (0–117)	11.5 (0–92)	15.5 (10–313)	22.5 (6–164)
Mann–Whitney	*p* = 0.0014	*p* = 0.0014	*p* = 0.0002	*p* = 0.0002

Data are presented as median (range).

When sIgY or cIgY were instilled prophylactically 30 min prior to the bacterial challenge, the cytokine levels in the bladder did not differ from the control group (Table 3).

**TABLE 3:** Prophylactic administration of IgY prior to bacterial infection does not significantly influence cytokine levels in the bladder.

	Concentration (pg/mL)			
	
	IL-1β	IL-6	CXCL2	G-CSF
sIgY (*n* = 9)	132 (0–452)	4 (0–28)	2 (0–138)	2 (0–46)
cIgY (*n* = 9)	164 (0–440)	16 (0–34)	18 (0–88)	12 (4–24)
Saline (*n* = 9)	0 (0–110)	1.5 (0–30)	4 (0–60)	7 (4–14)
Kruskal–Wallis	*p* = 0.12	*p* = 0.37	*p* = 0.65	*p* = 0.38

Data are presented as median (range).

## Discussion

Colonization of the urinary tract with *P. aeruginosa* can progress to infection and inflammation of the urinary tract (Tofte et al., 2017). In patients with transurethral catheters *in situ*, catheter-associated biofilms can often be prevented or treated by removing or replacing the catheters (Høiby et al., 2015). We aimed to establish a murine model of *P. aeruginosa* UTI without a permanent urethral catheter *in situ* and to test the potential of a promising non-antibiotic antibacterial strategy–avian anti-*pseudomonas* IgY antibodies–to prevent the development of UTI in this model. Anti-*pseudomonas* avian IgY antibodies were previously shown to prevent the onset of chronic *P. aeruginosa* lung infections in CF and to prolong the periods between exacerbations in CF (Nilsson et al., 2008). We hypothesized that instilling specific anti-*pseudomonas* IgY into the bladder might prevent the development of *P. aeruginosa* UTI and thereby reduce the need for antibiotic therapy in selected patients.

Previous murine UTI models differ in factors such as the inoculation routes, volumes, and concentrations, use of planktonic bacterial suspension or a permanent catheter (Hung et al., 2009; Conover et al., 2015). However, bacteria embedded in alginate resemble biofilm infections to a high degree (Christophersen et al., 2012, 2020). We hypothesized that the use of a temporary transurethral catheter instillation of *P. aeruginosa* alginate beads would establish a more stable infection compared to an injection of planktonic bacteria. This was not the case, as the success rate for the infection was higher in the planktonic model than in the alginate embedded bacteria model (Figure 2). This can probably partially be explained by bacterial adhesins being covered inside the alginate beads, thereby reducing the ability of the bacteria to stably attach to the bladder epithelium. Furthermore, the flow through the urinary tract is substantially higher than in a lung or wound environment (Ito et al., 2017), which could lead to a generally increased clearance of the injected bacteria or beads in our UTI model, compared to other infection models.

To improve the success rate of infection using planktonic *P. aeruginosa*, we tried to prevent post-infection bladder emptying by preventive induction of murine urinary void by massaging the abdomen before infection. Since complete bladder emptying pre-infection was impossible to ensure, we prospectively included a 30-min observation period after the procedure, to exclude the mice which urinated. It also proved to be important to initially inject an increased dose of anesthesia to avoid bladder emptying after the infection. With these technical improvements, we were able to successfully establish a chronic *P. aeruginosa* UTI without a permanent transurethral catheter. In further support of this, higher levels of the chemokine CXCL2 and the growth factor G-CSF, which are important for neutrophil recruitment, were measured in infected bladders compared to non-infected controls.

We evaluated relevant functional dosages of IgY by observing the growth retarding effect of sIgY and cIgY on *P. aeruginosa in vitro* (Figure 1). We found a dose-dependent effect of sIgY with 20% (4 mg/mL) IgY achieving the greatest reduction in bacterial growth for both bacterial strains (Figures 1A,C). High concentrations of cIgY had a residual effect on the serotype O:3 strain (Figure 1D). This is in accordance with the literature on the growth retarding effect of IgY on other pathogens (Lee et al., 2002; Zhen et al., 2008; Malekshahi et al., 2011; Wang et al., 2011). As 20% IgY was the highest concentration practically achievable and yielded the greatest reduction in bacterial growth, we chose to proceed with this concentration in our murine *in vivo* UTI model.

When injecting *P. aeruginosa* premixed with sIgY, the bacterial load in the bladder of IgY treated mice was lower for both inoculation concentrations compared to control mice, although this was only a trend with the high inoculation of 10^9^ CFU/ml (Figure 3). In some of the treated mice, IgY could prevent the detection of any bacteria after 20 h (Figure 3). In addition, the inflammatory cytokine response was almost completely absent in the sIgY-bacteria group, supporting the beneficial effect of sIgY in preventing cystitis (Table 2). We regarded this as a proof-of-concept that IgY binding to *P. aeruginosa* could increase the clearance of the bacteria from the urinary tract, even with high bacterial inoculi. With these results, we could proceed to a more clinically relevant approach of prophylaxis, focusing on a lower infective dose.

Surprisingly, we observed a significant prophylactic CFU-reducing effect of not only sIgY, but also non-specific cIgY (Figure 4). However, previously, in a series of *in vitro* and *in vivo* experiments using a *P. aeruginosa* lung infection mouse model, both sIgY and cIgY revealed activity, although the best effect was observed with sIgY (Thomsen et al., 2015, 2016a,2016b). This might be explained by the high abundance of IgY directed against numerous different antigens present in the yolk and the inherent polyclonal nature of the immunoglobulin eliciting a stronger immune response compared to mammalian IgG due to the phylogenetic distance between mammals/humans and chickens (Carlander et al., 2001; Ito et al., 2017). The residual antibacterial activity of cIgY that we and others observed could also serve as an explanation for why a multicenter international randomized control trial of prophylaxis with IgY was unable to demonstrate a difference in outcomes between sIgY and cIgY treated patients since the onset of chronic infection surprisingly was delayed by daily gurgling with both sIgY and cIgY (Schuster et al., 2019).

As the inflammatory response is an important part of the cystitis symptoms, we included the analysis of selected inflammatory markers to further characterize the anti-bacterial effect in our model. The most pronounced effect was observed when sIgY was co-installed with PAO1 intravesically. An almost complete abolition of the inflammation was observed in the sIgY group, strongly supporting the therapeutic potential of sIgY (Table 2). Further analysis showed a modestly increased inflammation of infected bladders as compared to non-infected bladders (Table 1). No statistical connection between the measured inflammatory markers and the observed primary endpoints was observed (Table 3). This may be explained by the relatively limited inflammatory response observed in the model, and also by the early timepoint chosen for evaluation.

Clinically, IgY has been demonstrated to have a broad application spectrum (Pereira et al., 2020) against bacterial infections, viral infections, fungi and parasites. The advantage of using IgY against a wide range of infections has been recognized and, as of now, several patents and clinical trials are on the way (Leiva et al., 2020). It has furthermore been acknowledged that IgY can either be used as a prophylactic or therapeutic treatment. In a gastrointestinal *Campylobacter jejuni* infection model in chicken, Tsubokura et al. (1997) found the prophylactic treatment with IgY to be more successful than therapeutic treatment.

Based on this, we find a substantial clinical potential of IgY for prevention of *P. aeruginosa* UTI, and probably also UTI caused by other frequent pathogens. At ICUs and other departments, patients carrying transurethral or suprapubic catheters, or nephrostomia patients, can receive intracatheter prophylaxis. Furthermore, patients with spinal cord lesions or other neurological defects, who are using intermittent catheterization due to impaired mictuation, might profit from IgY installation at the end of catheterization, and immediately before removal of the catheter. Potentially, IgY might also function as a therapeutic and might be able to potentiate antibiotic treatments. We have recently shown such an antibiotic-enhancing effect in our murine lung infection model, where IgY prophylaxis combined with azithromycin treatment achieved a significant reduction in lung bacteriology and inflammatory markers (Thomsen et al., 2021). The effect was believed to be mediated by the increased opsonization of bacteria, followed by phagocytosis, while the attracted neutrophils at the same time intracellularly accumulated azithromycin (Thomsen et al., 2021). High concentrations of azithromycin have been shown to be able to kill stationary phase *P. aeruginosa* (Imamura et al., 2005).

Our current study is limited by the short observation period of 20 h. However, as this project was focused on exploring potential prophylactic anti-*Pseudomonas* effects of IgY, we found it most important to observe for a clinically relevant period, as such a prophylactic strategy would probably be administered once or twice a day. In the future, we aim at expanding our model by repeated administration of prophylactic IgY prior to bacterial challenge, and to increase the time interval between prophylaxis and bacterial exposure, as this exceeded the scope of the current proof-of-concept study. We expect such a setup to be more complicated, as micturition cannot be sufficiently controlled in murine studies. A strategy to ensure that the prophylactic treatment, as well as the bacterial challenge remain in the bladder during a sufficient amount of time, must be identified, as well as acceptable, but still ethical, exclusion criteria in the case of urine excretion.

Even though using *P. aeruginosa* PAO1, which is originally a wound isolate, in a UTI model might not be the most obvious choice, we found this strain to be capable of inducing cystitis in our model. It is available worldwide and most commonly used in *P. aeruginosa* research. Furthermore, PAO1 was included in the strains used to vaccinate the hens for the sIgY production and is therefore appropriate to use in this proof-of concept study.

In conclusion, the present study showed an *in vivo* prophylactic CFU-reducing effect of installing specific avian anti-*Pseudomonas* IgY in the urinary bladder, against *P. aeruginosa* cystitis in a murine UTI model. The clinical perspectives of such new non-antibiotic applications are significant, especially since the product has already been developed for human use.

## Data availability statement

The raw data supporting the conclusions of this article will be made available by the authors, without undue reservation.

## Ethics statement

The animal study was reviewed and approved by Ethical Council for Animals, Ministry of Food, Agriculture and Fisheries of Denmark.

## Author contributions

FS, LC, KT, MJ, NH, and CM contributed to the conception and design of the study. FS, LC, SB, MP, IB, AL, and CM contributed to data acquisition, analysis, and interpretation. FS and CM wrote the first draft of the manuscript. All authors contributed to manuscript revision, read, and approved the submitted version.
